# Equity within AI systems: What can health leaders expect?

**DOI:** 10.1177/08404704221125368

**Published:** 2022-10-13

**Authors:** Emma Gurevich, Basheer El Hassan, Christo El Morr

**Affiliations:** 1 7991 York University, Toronto, Ontario, Canada.

## Abstract

Artificial Intelligence (AI) for health has a great potential; it has already proven to be successful in enhancing patient outcomes, facilitating professional work and benefiting administration. However, AI presents challenges related to health equity defined as an opportunity for people to reach their fullest health potential. This article discusses the opportunities and challenges that AI presents in health and examines ways in which inequities related to AI can be mitigated.

## Introduction

### AI and ML in health

Artificial Intelligence (AI) aims to imitate human intelligence and can be used to enable better decision-making processes in many areas including health. Machine Learning (ML) is a field of AI that aims to develop models for prediction and clustering. A ML algorithm uses a dataset to learn how to predict or cluster; this dataset is called the learning dataset.

When a ML model predicts a class to which a data instance belongs, the model is called a classifier; on the other hand, when the model predicts a number (e.g. age and number of months) it is called a regressor. Both classification and regression are part of a larger category called *supervised learning*. In supervised learning, the learning dataset contains the target or outcome (i.e. the dependent variable) of each instance in the dataset.

In the case of clustering, a ML algorithm aims to build a model that groups data into clusters based on a certain similarity measure among the data instances. It will then indicate the cluster to which each data instance belongs. The outcome is known in the learning dataset. When faced with new data, the clustering model chooses the cluster to which the new data belongs. Since the outcome of the new data instance is not known in the learning dataset, clustering is said to belong to *unsupervised learning*.

The application of AI and ML in healthcare is expanding. AI has been proven to be successful in early diagnosis, early detection, prediction, and choosing between treatment alternatives,^1^ in medical imaging interpretation and processing, in pathology, gastroenterology, and ophthalmology, to name a few domains.^2^

### Health equity

Equity is defined as fairness and justice for all. Health equity is a principle dedicated to maximizing people’s health potential and reducing health disparities. Hence, health equity considers people’s social factors, also known as the Social Determinants of Health (SDoH), as determinants of their ability to equitably access health. SDoH are known to affect individual and population health with ample evidence indicating that poor health is directly related to social factors.^3^ In Canada, SDoH include Aboriginal status, race, disability, early life development, education, sexual orientation, social exclusion, social safety net, unemployment and job security, employment and working conditions, food insecurity, health services, gender and gender identity, housing, income, and income distribution.^4^ Canada is a multicultural society, and racialized populations include South Asians, Chinese, and Black communities. Racialized Canadians’ physical, mental, and social health are due to experience of lower rates of income, higher rates of unemployment, and lower occupational status.^4^

While the implementation of AI in health has potential benefits, AI can also undermine health equity.^2^ The objective of this paper is to assess the interplay between equity and AI.

## Equity in health: AI potential benefits

AI has a high potential in transforming decision-making and medical treatment, specifically, in primary care.^5,6^ Many vulnerable populations access healthcare services through primary care; AI systems in these settings can have a positive impact on vulnerable populations.^7^

AI solutions have proven to be beneficial for patients in areas of clinical oncology, dermatology, the prediction of postpartum depression, the diagnosis of diabetic retinopathy in youth, and in the management and nutrition counselling for patients with diabetes and other chronic diseases.^6,8-14^ AI has also been emerging in preventative care^15,16^ and the medical robot sector.^17-19^ Furthermore, AI-assisted medical services can benefit underserved rural areas.^20^ Currently, initiatives have been implemented to properly manage health systems, track interactions, improve cost-efficiency, and to effectively increase well-being.^21^

In addition, patient-centred care is expected to be positively impacted by AI applications, specifically in communication with patients. Many patients in healthcare settings have limited English proficiency and, as a result, may suffer from a larger number of medical complications.^22^ AI can play a role in overcoming language barriers. Indeed, AI-based applications have been developed for patients to choose their preferred language through standardized instructions.^23^ The list of areas in healthcare that can benefit from AI, including individual and public health, is endless.^24-37^

Not only can AI potentially enhance health equity by improving healthcare provision, but it also has potential to help overcome human decision-making, which is often clouded by biases (including cognitive bias); for instance, AI-based systems have helped in reducing the number of incorrectly denied refugee claims.^38^

## Equity in health: Potential AI concerns

While AI has great potential in enhancing health equity, there are concerns related to its use in healthcare. It is imperative that AI initiatives do not continue perpetuating the same inequities already faced by vulnerable individuals.^6,7,9,11-13,37,39,40^ For instance, an AI application that aimed to predict how likely an individual is to recommit a crime was proven to be substantially biased against Black people as it consistently predicted that they were at a high risk of recommitting a crime in comparison to White individuals. However, statistics show that they were only half as likely to recommit a crime as their White counterparts.^7^ This software reflects inherent and explicit social biases surrounding race. The same risk applies to Canada, while race correction is used in kidney and lung function measurements, for example, variation exists within the healthcare professionals’ body.^41^ As LLana James, AI, Medicine and Data Justice Post-Doctoral Fellow at Queen’s University puts it: “Race-medicine is not solely about Black people, it is also about how White people have used themselves as a primary reference in clinical assessment, and have in so doing, not necessarily tended to the precision of the science.”^41^ AI models trained on past data will reflect the data biases.

Other instances of unfairness towards vulnerable groups have been reported across algorithms used for medical management, public health, and federal compensation programs.^12^ Health data used to train algorithms is often collected from a mostly White population, and/or excludes ethno-racial information altogether; the resulting models may be biased against Black, Indigenous, and People of Colour (BIPOC). On the other hand, historically, when ethno-racial data has been included, it has been incorporated inappropriately. For instance, pulmonary function and pain scores that are adjusted for race continue to be used throughout the healthcare systems contributing to poor health outcomes for People of Colour.^12^

These are a few examples of SDoH impact on AI algorithms, the main lesson is that those with privilege (i.e. White people, men, higher socioeconomic status, and English speaking) tend to have better outcomes with the use of these algorithms as opposed to those with less privilege (i.e. women, non-binary folks, BIPOC, and English as a second language); hence, the need to mitigate algorithmic biases.

Building ML models based on biased tools only exacerbates bias. Biases in such algorithms reflect historical influence that encapsulates systemic racism, sexism, and other types of socioeconomic biases. This often occurs due to over-/under-representation of specific populations in training data sets, or due to the implicit biases of those creating the algorithms. This is later reflected in the predictive power of algorithms. Undesirable biases further perpetuate existing health inequities, putting vulnerable populations at a greater risk of experiencing poor health outcomes.^7^ There is a need to train AI and ML algorithms to be inclusive so that biases are addressed.^11^

## Lesson for health leaders: Mitigating AI inequities

AI solutions can only be as successful as their benefits; it is imperative that the disadvantages of such technologies and their potential pitfalls are mitigated. Despite this, it should be noted that inequalities exist in access to AI technology, as well as unfairness in who it may provide an advantage and disadvantage to.^39^

### Equity assessment

The largest concern surrounding AI solutions is the potential for systems to continue perpetuating inequities.^6-8,11,12,39,40^ Thus, AI initiatives should have two main goals: (1) they should be designed and utilized in a manner that does not create or maintain health disparities currently experienced by vulnerable groups, and (2) they should address and remove existing health disparities.^6,39^

To ensure that all healthcare-based AI embodies these two goals, it is important to create system level changes such as a federal and/or provincial regulatory framework that oversees the equity dimensions in the implementation of AI solutions.^12,39^

The Federal Drug Agency (FDA) in the United States has introduced a regulation for AI applications that are designed for use in clinical decision-making or for inpatient health data analysis or medical imaging. This step forward is still limited as it leaves a myriad of applications designed for other purposes (e.g. resource allocation and access to public health) and affecting patients and healthcare delivery from regulations.^39^ It is our view that such applications should also be regulated. Currently, the government of Canada is tabling Bill C-27 that will enact the Artificial Intelligence and Data Act (AIDA) “to regulate international and interprovincial trade and commerce in artificial intelligence systems by requiring that certain persons adopt measures to mitigate risks of harm and biased output related to high-impact artificial intelligence systems.”^42^ While it is not enacted yet, it addresses assessment, mitigation, and monitoring obligations; it has a provision to establish measure “to identify, assess, and mitigate the risks of harm or biased output that could result from the use of the system.”^42^ The definition of a “high-impact system” is not clear yet and is left to be established in AIDA section 5(1). It is yet to be discovered how the law will impact the Canadian innovation and application landscape. A regulatory body for AI applications will probably take shape on the provincial and territorial levels; however, some levels of coordination and collaboration among national, provincial, and territorial entities would be expected. For AI applications intended for health, a regulatory body would collect evidence from available research, and might recommend or require (1) AI-reporting based on current recommendations in the field,^43,44^ especially those related to AI-equity and AI-interpretability,^45,46^ as well as (2) submission of specific evidence (i.e. randomized control trial).^47^

### Equity at the core of AI projects

It is important to incorporate an equity dimension in the different stages of AI creation, from assessing the representativeness of data, to continuous surveillance of systems after deployment.^12^ In the development stage, for example, it is imperative that data used in the training of predictive algorithms includes ethno-racial, sex, and gender characteristics as there are apparent differences in the risk factors for certain diseases and health outcomes based on these factors.^9,11^ This in turn will reduce the chance of a distributional shift, a phenomenon where the training data is not representative of the population.^11^

Likewise, it is important to disclose the distribution of factors that are not routinely reported as these may increase desirable bias while exposing undesirable biases.^9,11^ Moreover, one should report limitations related to the training data set (e.g. ethno-racial).^11^ Furthermore, there is a need to validate models using data samples other than retrospective data as these may not fully capture biases.^12^ When implementing the model, it is good practice to make certain that systems undergo continuous evaluation,^12^ to ensure that models can perform as designed and work to remove existing systemic inequalities within the healthcare system.^6^

### Involving stakeholders

The implementation of AI in projects must be a collaborative effort. It should include physicians, patients, and communities from diverse backgrounds of social, cultural, and economic contexts. One way to involve recipients of care is by using Patient-Reported Outcome Measures (PROMs) to understand health-related outcome measures from a patient's perspective. Furthermore, engaging patients and their communities with Information Technology (IT) teams that produce the algorithms can help assess and address AI bias. In this context, training and education on health equity is important for IT teams to understand the potential effects of AI initiatives on health equity.^48^

### Algorithmovigilance

Due to the number of systemic inequities and health disparities, developing and testing algorithms that allow systematic surveillance and vigilance in the development of AI models in healthcare becomes important. Algorithmovigilance involves algorithms’ evaluation and monitoring to prevent AI bias and, thus, must be part of AI projects. Debiasing steps can be taken within a project as well. For instance, debiasing can be the result of retraining models without race variables (fairness through unawareness) or measuring the differences in outcomes between privileged and unprivileged groups.^49^

### Need to address SDoH

The use of AI is emerging in public health; however, it faces multiple challenges from a social justice perspective. Challenges include focusing on data while drawing the attention away from the causes of health inequities such as the SDoH. AI intended for social good that neglects this aspect may create new vulnerabilities and fail to attain the projects’ aim. Employing SDoH lens in AI initiatives will benefit the public and help create a digital world oriented toward social justice and health equity.^50^

### Need for data regarding social context

AI technologies and advanced analytics are being integrated into healthcare to make key clinical decisions. Thus, AI technologies must be provided with data related to social contexts, otherwise the work produced will be short of considering health equity, especially in primary care. In one example, AI models were used in a primary care setting, 20% of patients preferring to use Spanish were misclassified as preferring English due to imbalanced training.^48^ Integrating lived experiences of diverse communities is key to increasing equity of AI models.

### Challenges specific to the Canadian context

One limitation is the cost of implementing AI in northern and remote communities as the number of people using the AI-based application is significantly lower. However, AI-based may produce cost savings, if the AI-related cost could be balanced by cost savings is something to be studied. There is also a challenge regarding Indigenous health practices and values, as these are different from medicine as practiced in the healthcare system. AI models based on Indigenous practices and values would be needed for an Indigenous population practicing medicine. Moreover, given the multicultural environment in the Canadian society, culturally inclusive AI models that respect the variety of culture could be needed and would be need to be designed.^51^

A large portion of AI research and development in Canada is a part of the Pan-Canadian AI Strategy which is directed by the Canadian Institute for Advanced Research (CIFAR). CIFAR partners with the following institutions: Alberta Machine Intelligence Institute (AMII), Montreal Institute for Learning Algorithms (MILA), and Vector Institute to bring together researchers from across the country.^52^ While CIFAR involves AI in multiple areas, health-related projects include understanding how gene interactions impact health and development as well as the effect of human microbiomes on health, development, and behaviour. Ethical implications of AI in health would be important to research with these institutions.

### Important considerations

It is critical for policy-makers to understand that bias mitigation should not end with AI model development but, rather, extend across the product lifecycle. We believe in line with Thomasian et al.^53^ that the following considerations are key for future development of equitable AI for health:

1) *Bias alleviation during model development*
• Study how the quality and availability of equity related data (e.g. immigration, race, and gender) can impact model performance.
• Assemble and organize open databases with non-identifiable patient information to overcome imbalance in equity related data.
• Make use of collaborative model training (e.g. federated learning and cyclic weight transfer) as they can increase data size without transferring patient data between health organizations.
2) *Bias mitigation of the machine learning model*
• Consider non-routinely reported factors, such as socioeconomic status and race, when developing models, especially when the models are intended to serve in areas where inequity is well documented.
• Use appropriate bias metrics selected based on the algorithm's objectives.
3) *Post deployment validation*
• Validate the model prospectively and not only based on retrospective datasets. This is important as models trained on retrospective data alone might behave differently (e.g. cause harm) when new instances emerge in real-life.
4) *Auditing for interpretability and bias*
• Audit for equity/biases continuously throughout implementation.
• Audit for interpretability of the models to avoid unintended consequences of technology and mitigate human factors/errors post implementation.

## Conclusion

While AI can handle the complex and multidimensional fabric of the Canadian population and deal with big data, it cannot do so unless trained to do it. Hence, mitigation of AI potential biases is needed; particularly, processes and frameworks to follow during the design, and quality monitoring processes are important to implement.

It is important to note that AI has proven to be cost effective in many cases. For example, autonomous AI was as effective for and less costly (up to $34 for compared with telemedicine and $64 and $91 compared with ophthalmoscopy) for retinopathy of prematurity screening.^54^ Also, research shows that AI-based tools produce cost savings if used as a strategy in screening colonoscopy,^55^ and for breast cancer screening.^56^ While this is encouraging, it cannot be generalized and need to be studied on a case by case basis.^57^

Equity as an aim in healthcare delivery is an important and often overlooked factor in health informatics. AI can provide potential benefits and risks to patients as it can enhance or diminish equity. While steps to mitigate equity concerns in AI projects are needed and available, a systematic equitable AI approach is yet to be developed.

While it is likely that the proper inclusion of SDoH will require more work on the side of the creators of algorithms (and will be more resource intensive), the cost implications of disregarding the SDoH within the current healthcare system are also high and defeat the very purpose of the healthcare system. The Canadian healthcare system would benefit from implementing SDoH informed AI solutions in order to prevent health incidents and provide an equitable access to health.

It is our view that health leaders need to support the inclusion of SDoH within Canadian healthcare in general and particularly the expected upcoming wave of AI-based systems. Simultaneously leaders should advocate for the inclusion of equity in AI projects and support the inclusion of anti-racism and anti-oppressive practices in the healthcare industry.
